# Supplementary material for: Sustainable Green Processing of Grape Pomace Using Micellar Extraction for the Production of Value-Added Hygiene Cosmetics
Source: Molecules. 2022 Apr 10;27(8):2444. doi: 10.3390/molecules27082444 (PMC9025557; doi:10.3390/molecules27082444)
Supplement: Supplementary file 1 [file molecules-27-02444-s001.zip › molecules-1646885-supplementary.pdf]

Table S1. Optimized parameters for MS/MS transitions, declustering potential (DP), entrance potential (EP), collision cell exit potential (CXP) and collision energy (CE).

Compound	Precursor ion [m/z]	Main product ions MS^2 [m/z]	Ionization mode	DP [V]	EP [V]	CE [V]	CXP [V]
tartaric acid	148.9	87.0	ESI -	-25	-10	-18	-5
tartaric acid 1	148.9	73.0	ESI -	-25	-10	-26	-5
maleic acid	114.9	70.9	ESI -	-55	-10	-14	-7
maleic acid 1	114.9	45.0	ESI -	-55	-10	-22	-5
DL-malic acid	132.9	114.9	ESI -	-35	-10	-16	-7
DL-malic acid 1	132.9	71.0	ESI -	-35	-10	-20	-5
gallic acid	168,9	124,8	ESI -	-40	-10	-20	-7
gallic acid-1	168,9	78,9	ESI -	-40	-10	-28	-7
D-(-)-quinic acid	190,9	84,9	ESI -	-75	-10	-28	-7
D-(-)-quinic acid-1	190,9	93,0	ESI -	-75	-10	-30	-5
quercetin	300,9	151,0	ESI -	-90	-10	-30	-7
quercetin 1	300,9	179,0	ESI -	-90	-10	-26	-7
(+)-Catechin	290.9	139.0	ESI +	81	10	23	10
(+)-Catechin 1	290.9	123.0	ESI +	81	10	47	10
(-)-Epicatechin	290.9	139.0	ESI +	81	10	23	10
(-)-Epicatechin 1	290.9	123.0	ESI +	81	10	47	10
(-)-Catechin 3-gallate	306.9	288.8	ESI +	71	10	21	10
(-)-Catechin 3-gallate 1	306.9	163.0	ESI +	71	10	11	12
L-methionine	150.0	103.9	ESI +	1	10	15	8
L-methionine 1	150.0	132.9	ESI +	1	10	13	10
L-tryptophan	205.1	188.0	ESI +	1	10	15	8
L-tryptophan 1	205.1	145.9	ESI +	1	10	25	12
D-(-+)-xylose	149.9	104.0	ESI -	-55	-10	-10	-7
D-(-+)-xylose 1	149.9	89.9	ESI -	-55	-10	-10	-7
sucrose	340.9	179.0	ESI -	-90	-10	-20	-7
sucrose 1	340.9	88.9	ESI -	-90	-10	-26	-7
